# Reduced Connexin26 in the Mature Cochlea Increases Susceptibility to Noise-Induced Hearing Loss in Mice

**DOI:** 10.3390/ijms17030301

**Published:** 2016-02-26

**Authors:** Xing-Xing Zhou, Sen Chen, Le Xie, Yu-Zi Ji, Xia Wu, Wen-Wen Wang, Qi Yang, Jin-Tao Yu, Yu Sun, Xi Lin, Wei-Jia Kong

**Affiliations:** 1 Department of Otorhinolaryngology, Union Hospital, Tongji Medical College, Huazhong University of Science and Technology, Jiefang Avenue 1277, Wuhan 430022, China; star1028@126.com (X.-X.Z.); cs88030067@sina.com (S.C.); leoxie5842@gmail.com (L.X.); jiyuzi1987@sina.com (Y.-Z.J.); wuxia027@163.com (X.W.); wangwenwen@hust.edu.cn (W.-W.W.); yangqitj@sina.com (Q.Y.); yujintaoly@163.com (J.-T.Y.); 2 Department of Otolaryngology Head and Neck Surgery, Emory University School of Medicine, 615 Michael Street, Whitehead Bldg Rm#543, Atlanta, GA 30322, USA; xlin2@emory.edu; 3 Institute of Otorhinolaryngology, Tongji Medical College, Huazhong University of Science and Technology, Wuhan 430022, China

**Keywords:** Connexin26, GJB2, noise-induced hearing loss

## Abstract

Connexin26 (Cx26, encoded by *GJB2*) mutations are the most common cause of non-syndromic deafness. *GJB2* is thought to be involved in noise-induced hearing loss (NIHL). However, the role of Cx26 in NIHL is still obscure. To explore the association between Cx26 and NIHL, we established a Cx26 knockdown (KD) mouse model by conditional knockdown of Cx26 at postnatal day 18 (P18), and then we observed the auditory threshold and morphologic changes in these mice with or without noise exposure. The Cx26 KD mice did not exhibit substantial hearing loss and hair cell degeneration, while the Cx26 KD mice with acoustic trauma experienced higher hearing loss than simple noise exposure siblings and nearly had no recovery. Additionally, extensive outer hair cell loss and more severe destruction of the basal organ of Corti were observed in Cx26 KD mice after noise exposure. These data indicate that reduced Cx26 expression in the mature mouse cochlea may increase susceptibility to noise-induced hearing loss and facilitate the cell degeneration in the organ of Corti.

## 1. Introduction

*GJB2* mutations are the most common causes of non-syndromic deafness and more than 100 *GJB2* mutations are linked with hearing impairment. Gap junctions (GJs), which facilitate the exchange of ions, small molecules and second messengers, are arrays of intercellular channels that are composed of connexin protein subunits [1,2,3,4]. In the mammalian inner ear, connexin26 (Cx26), which is encoded by *GJB2*, is mainly assembled with Cx30 to form heteromeric Cx26/Cx30 gap junctions in supporting cells and fibrocytes [4,5]. Cx26 is thought to participate in potassium circulation, calcium propagation or endogenous signal communications in the cochlea.

Due to the vital role of Cx26 in cochlear physiology, numerous studies have explored the correlation between the genetic basis of *GJB2* mutations/polymorphisms and other types of hearing loss, such as noise-induced hearing loss (NIHL); however, the results have been contradictory. The *GJB2* 35delG mutation is the most common mutation detected in the *GJB2* gene in the Caucasian population [6,7]. According to previous studies, the 35delG mutation can lead to a frameshift and premature termination of protein translation. Therefore, persons homozygous for the 35delG mutation may suffer mild to profound hearing loss, but carriers may have normal hearing [8,9]. In 2004, an investigation was conducted among Swedish workers to determine whether *GJB2* 35delG mutation carriers had high susceptibility to NIHL, and the results demonstrated that there was no significant correlation between the *GJB2* 35delG mutation and NIHL [10]. Similar conclusions were drawn in Polish and Brazilian population [11,12]. However, another analysis of single nucleotide polymorphisms (SNPs) indicated that the *GJB2* gene may be significantly associated with NIHL. In this study, 119 matched pairs of Polish workers who were sensitive or resistant to noise were included, and the odds ratio (95% CI) of the *GJB2* SNP (rs3751385) was discovered to be 2.064 [13]. Recently, another study on Chinese NIHL workers suggested that gene-gene interaction among *GJB2* SNP (rs137852540), SOD2 and CAT might account for NIHL development; however, when analyzed independently, the single *GJB2* SNP (rs137852540) did not increase the risks of NIHL [14].

To explore the association between *GJB2* and NIHL, more objective animal models should be established. To avoid having to breed different mutation models, the conditional gene knockout mouse is an ideal and convenient tool that may be useful for hearing research. The loss of Cx26 in the cochlea may partially imitate the loss of function caused by corresponding mutations. However, previous Cx26 null mice exhibited congenital severe hearing loss after birth when Cx26 was reduced during embryonic periods. These mice were good models for congenital non-syndromic deafness but were not appropriate for noise exposure [15,16,17]. Our group and Zhao *et al.* [18,19,20] had successfully induced Cx26 knocked down (KD) in mice at postnatal day 10 to12 (P10 to P12), in which hearing impairment was discovered to be mild. Subsequently, a series of deletion time points before or after the onset of hearing (about P14) were systematically investigated in the similar model. The results suggested that the hearing might not be affected up to one month following deletion of Cx26 that occurs after P16 [21]. Therefore, we predict that Cx26 reduction at more mature stages will provide a relative safe time for noise exposure research.

To further study the correlation between Cx26 and NIHL, herein, cochlear Cx26 was reduced at postnatal day 18 (P18) in animals that were exposed to discontinuous white noise. Our data suggests that normal hearing is maintained in mice when Cx26 is reduced in mature cochlea; however, loss of Cx26 exacerbates hearing loss and the cochlear cell degeneration that occurs after acoustic trauma.

## 2. Results

### 2.1. Connexin26 Deletion in Conditional Cx26 Knocked down Mice

**Figure 1:**
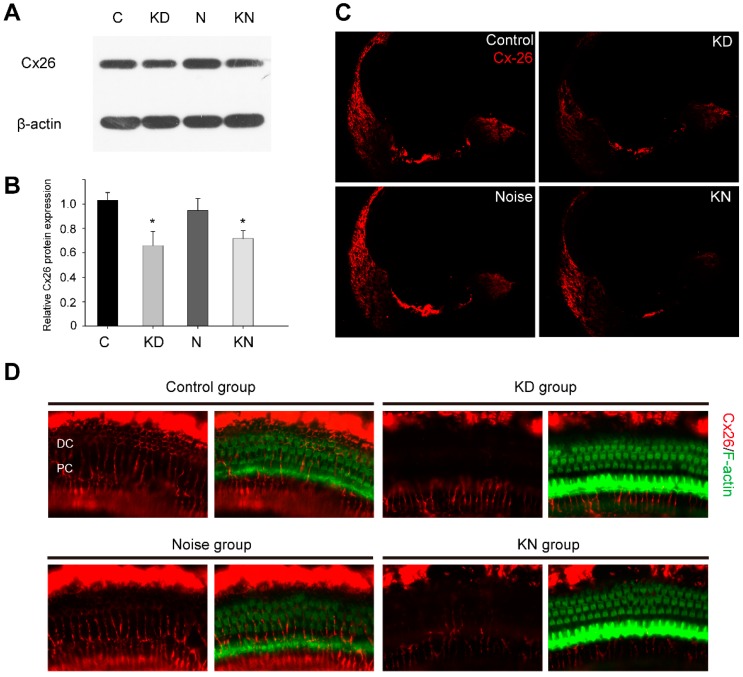
Connexin26 deletion in conditional connexin26 (Cx26) Knockdown (KD) mice after noise exposure. Summary of Cx26 expression in control and experimental groups. (**A**,**B**) Western blot and histogram detecting Cx26 in the cochleae of control, KD, noise and knockdown+noise groups (KN groups) at P30 (*n* = 4 in each group); (**C**) Immunolabeling of Cx26 (**red**) in the cochlear sections of control and different experimental groups at P30; (**D**) Immunolabeling of Cx26 (**red**) and phalloidin (**green**) in flattened cochlear preparations of control and experimental groups at P30. There was an obvious Cx26 reduction in Deiter and Pillar cells of KD and KN mouse groups. Abbreviations: PC: Pillar cell; DC: Deiter cell. * *p* < 0.05 when compared with the control group.

In this study, Cx26 was successfully knocked down in mice cochleae at P18. Compared to the control group at P30 (Figure 1A,B), western blots indicated that the Cx26 protein in KD, noise and KN groups were 66.0% ± 11.5% (*p* = 0.014), 94.8% ± 9.9% (*p* > 0.05) and 71.8% ± 5.7% (*p* = 0.031), respectively. As shown in Figure 1C, Cx26 staining (red) was detected in the organ of Corti, lateral wall and spiral limbus in control and noise groups at P30, and the labeling was partly absent in the organ of Corti and spiral ligament in KD and KN group. Following investigation of flattened cochlear preparations, the Cx26 staining (red) was restricted to the circumference of the Pillar and Deiter cells in the control and noise group. In contrast, there was significantly reduced Cx26 staining of these cells in the KD and KN groups (Figure 1D).

### 2.2. Hearing Thresholds Shift after Cx26 Reduction and Noise Exposure

**Figure 2:**
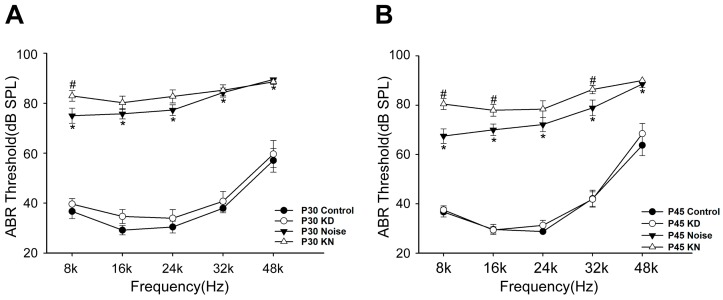
Hearing thresholds shift after Cx26 reduction and noise exposure. Auditory thresholds were measured in control, KD, noise and KN group at P30 (**A**) and P45 (**B**). * Significantly different from the control group (*p* < 0.01); ^#^ Significantly different from the noise group (*p* < 0.01). Plot legends for different groups are given in the panel.

Cx26 reduction could potentially exacerbate hearing loss in the KN group. In previous studies, knocking down Cx26 in mature cochlea (after P16) could not lead to a rapid and notable deafness. Additionally, the mice showed a stable hearing one month following the knockdown, providing a prerequisite for noise exposure in the present study. In the present, there were no substantial hearing differences between control and KD group at P30 and P45 (Figure 2A,B). At P30, the hearing thresholds in the noise group at 8–48 kHz were 75.0 ± 2.99, 75.8 ± 2.1, 77.25 ± 2.15, 84.25 ± 1.80 and 89.5 ± 1.71 dB SPL, respectively. The ABR thresholds in the KN group were 82.9 ± 2.13, 80.2 ± 2.64, 82.7 ± 2.59, 85.2 ± 2.19 and 88.5 ± 1.08 dB SPL, respectively. After two weeks, the auditory threshold at low frequency showed partial recovery in noise group (Figure 2B); however, recovery was not found in KN group. At P45, the thresholds at 8–48 kHz in noise group were 67.5 ± 2.94, 70.0 ± 2.34, 72.1 ± 2.84, 78.9 ± 3.16 and 88.6 ± 1.37 dB SPL, respectively. However, the thresholds in the KN group were 80.5 ± 2.22, 78.0 ± 2.40, 78.4 ± 3.40, 86.4 ± 1.69 and 90.0 ± 0.00 dB SPL, respectively. The differences between noise and KN group were significant at 8, 16 and 32 kHz (*p* < 0.01, student *t*-test).

### 2.3. Cx26 Reduction Exacerbates Hair Cell Loss in the Knockdown + Noise Groups (KN Groups)

**Figure 3:**
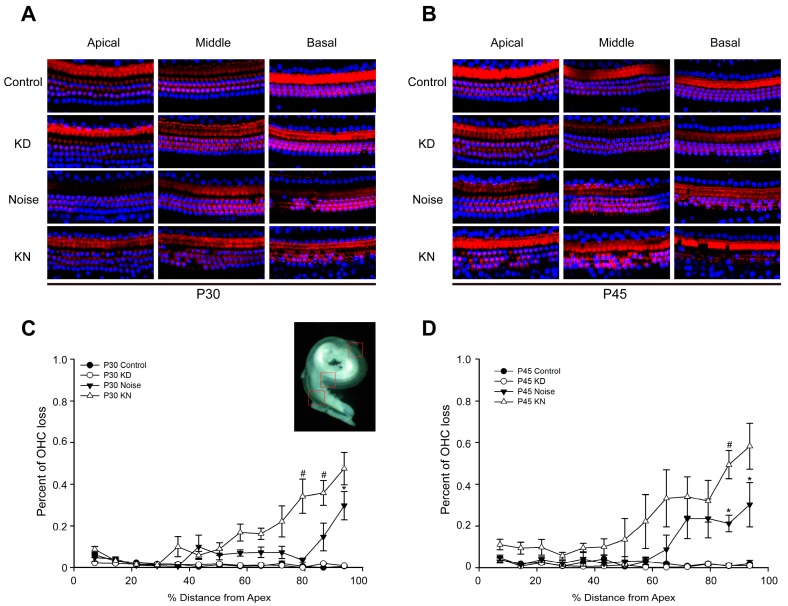
Hair cell loss patterns in control, KD, noise and KN groups. Patterns and time courses of OHC loss in control and experimental groups (*n* = 3–4 in each group). Panels (**A**,**B**) show the examples of the HC nucleus (**blue**) and *F*-actin (**red**) in control, KD, noise and KN groups; Three rows of OHCs are at the bottom; Panels (**C**,**D**) show quantifications of OHC loss at specific cochlear locations in control and different experimental groups. The inset in panel C shows a dissected surface preparation and the red frames represent different check points.* Significantly different from the control group (*p* < 0.05); ^#^ Significantly different from the noise group (*p* < 0.05). Plot legends for different groups are given in the panels.

At P30 or P45, no substantial hair cell (HC) loss was observed in control and KD groups (Figure 3A,B). However, obvious outer hair cell (OHC) loss was observed in the basal region in noise and KD groups. At P30, 4%–30% of outer hair cell loss was found in the cochlear middle to basal turn of the noise group (Figure 3C), and the stereocilium and cuticular plate (phalloidin staining, red) were degenerated in the corresponding region. The outer hair cell loss in the KN group was more severe than that in the noise group. The degeneration of OHCs was scattered in middle turn and some focal lesions could be seen in the basal turn (Figure 3A,C). At P45, basal OHC loss in noise and KN groups was exacerbated. Hair cell counting indicated that approximately 10.0%–58.2% of the OHCs were lost in the cochlear middle to basal turn of the KD group (Figure 3B,D).

### 2.4. Cell Degeneration Pattern after Cx26 Reduction and Noise Exposure

**Figure 4:**
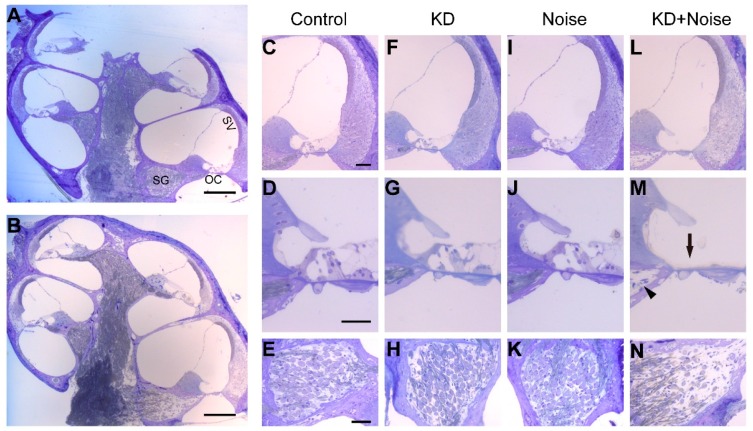
Cochlear morphology of the basal turn in control, KD, noise and KN groups. A full view of a cochlea obtained from noise (**A**) and KN groups (**B**); Panels **C**, **F**, **I** and **L** show the morphology of the cochleae at the basal turn in control and experimental groups; Panels **D**, **G**, **J** and **M** show the morphology of the magnified OC in panels **C**, **F**, **I** and **L**, respectively; Panels **E**, **H**, **K** and **N** show the SGN at the basal turn in the different groups. Abbreviations: SV: stria vascularis; OC: organ of Corti; SG: spiral ganglia. The scale in panel represents 200 μm, and scales in panel **C**, **D** and **E** represent approximately 40 μm.

Resin sections were stained for morphological observation at P45 (Figure 4). The panel A and B show a full view of a cochlear section in the noise and KD group, respectively (Figure 4A,B). The basal turns in the four groups were magnified in the right panels. Neither cell degeneration nor deformity were obvious in the organ of Corti (OC), stria vascularis (SV) or spiral ligament of control and KD groups (Figure 4C,F). The tunnel of Corti (TC) and the Nuel’s space were well developed in control and KD group (Figure 4D,G). In contrast, OHC loss was obvious with a general preservation of surrounding supporting cells in the basal turn of noise group (Figure 4J). Further, focal collapse of basal OC (Figure 4M, black arrow) in KD group was observed and the nerve fibers (Figure 4M, black arrowhead) were missing in some sections; however, the OC was generally preserved in the middle and apical turn (Figure S1). Consequently, the spiral ganglion neuron degeneration in corresponding region was observed (Figure 4N). Some enlarged intercellular spaces were observed in the spiral ligament of the KN group (Figure 4B); however, significant differences were not observed in stria vascularis (SV) when comparing between the four groups using an optical microscope.

### 2.5. Ultrastructural Changes after Cx26 Reduction and Noise Exposure

**Figure 5:**
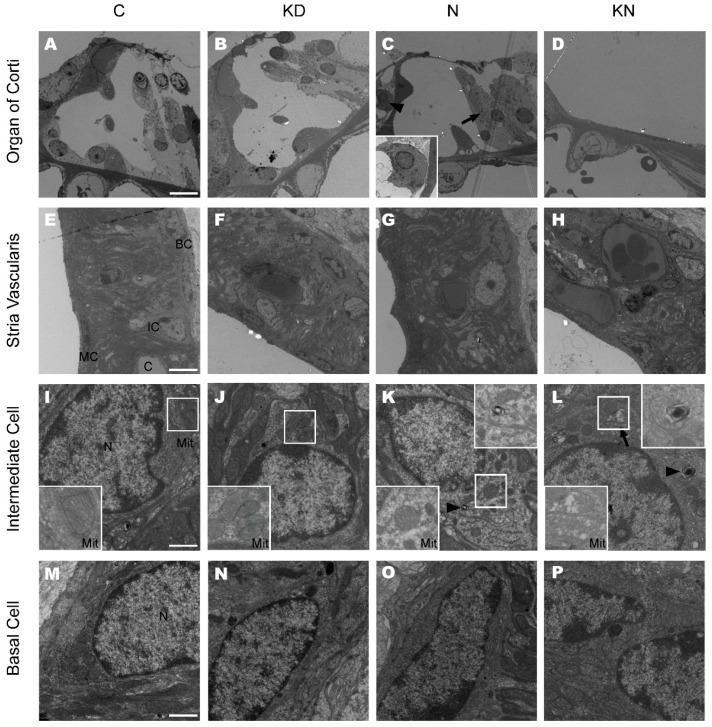
Ultrastructure of cochleae at the basal turn in control, KD, noise and KN groups. The ultrastructure of the basal turn of the cochlea was observed in control and different experimental groups at P45 (*n* = 3 in each group). Panels **A**, **B**, **C** and **D** show the basal OC in control, KD, noise and KN groups. Distinct OHC loss was observed in noise group (C) and the basal OC was collapsed in KN group (D). The inset (panel **C**) in the left bottom show the inner hair cell; Panels **E**, **F**, **G** and **H** show the SV at the basal turn in different groups; Panels **I**, **J**, **K** and **L** show the ultrastructure of IC at the basal turn in control and experimental groups. The insets (panels **I**, **J**, **K** and **L**) on the left bottom show mitochondria and the myelinbody was magnified in the upper right boxes (Panel **K** and **L**). Panels **M**, **N**, **O** and **P** show the ultrastructure of BC in stria vascularis. Abbreviations: SV: striavascularis; OC: organ of Corti; IC: intermediate cell; BC: basal cell; MC: marginal cell; C: capillary. The scales in panel **A**, **E**, **I** and **M** represent 10, 5, 1 and 1 μm, respectively. The arrow in **L** is pointing out a mitochondria and the arrowhead indicates a myelinbody.

The OC and SV were observed at P45 by a transmission electron microscope. As shown in Figure 5A,B, the TC and Nuel’s space were well developed in the control and KD group. The Pillar cells and Deiter cells were intact and no significant mitochondria pathological lesions were found in these two groups. However, in the noise group, outer hair cell degeneration was distinct and some Deiter cells were transitioning into tall columnar cells that support the cuticular plate (Figure 5C, arrow). Some of the inner hair cells in the noise group displayed a shrunken nucleus and a dark cytoplasm with different vacuoles (Figure 5C, arrowhead). As shown in optical microscope, the basal OC from some sections was totally collapsed in the KN group. Massive loss of hair cells and supporting cells occurred in the KN group at P45 (Figure 5D).

The structure of the SV did not display significant differences among the four groups. Additionally, the three-layers structure was well arranged and the strial capillaries were opened with normal vascular endothelial cells in the four groups (Figure 5E–H). Marginal cells (MC) extending long processes filled with mitochondria were found in the SV. A few swollen mitochondria with disrupted cristae could be observed in the processes of MC in the KN group. A majority of mitochondria with distinct cristae were observed in perinuclear cytosol of intermediate cells (IC) from control, KD and noise group (Figure 5I,J,K). In the noise group, the myelin body was seen occasionally in intermediate cell (Figure 5K arrowhead). In KN group, some mitochondria with disrupted cristae could be found and a few myelin bodies still existed (Figure 5L). No obvious ultrastructural lesions were found in basal cells of the four groups (Figure 5M–P).

## 3. Discussions

Cx26 mutations are the most common cause for non-syndromic deafness [22]. Various deafness mechanisms and explanations have been proposed by studying Cx26 null mice. In embryonic Cx26 null mice, a majority of hair cell loss in the middle and basal turn and SG degeneration in corresponding regions have been observed at P30 and are thought to cause severe congenital deafness [16,23]. In comparison with other transgenic mouse strains, OC deformity with a closed tunnel of Corti have become the focus and have provided new evidence in recent studies [15,17]. By use of conditional knockout mice, Cx26 could be deliberately knocked down at different time points. Developmental disorders of the mouse cochlea have been elaborately investigated, and the results indicate that reducing Cx26 at embryonic periods or during early postnatal days could arrest TC development [18,24]. More importantly, hearing loss was alleviated and led to later-onset deafness when the knocking down time points came at later days after birth (P10–P12) without obvious developmental disorders [18,20,21]. Although Cx26 played an indispensable role in cochlear postnatal development, little evidence on Cx26 functions in mature cochlea was found. A previous mechanism involving the endocochlear potential (EP) or propagation of calcium still could not explain that reduced widespread Cx26 in later postnatal development stages (P10–P12) only leads to mild hearing loss [18,20,25,26].

Mice cochleae is born immature and the normal hearing matures at P16–P18 [27,28]. Zhao’s group has observed significant hearing loss at P35–P60 by deleting Cx26 only in outer Pillar and Deiter cell regions at E18 [29]. In this study, we reduced the cochlear Cx26 at P18 to avoid the influence on the cochlear development and a significant Cx26 reduction could be ensured in OC region. Compared with control siblings, there were no significant differences in the ABR thresholds, hair cell number or ultrastructure in KD mice at P45. Similar results were observed by Lin’s group that the mice deleting Cx26 at P16 and P30 showed a normal hearing in the following month [21]. These results indicate that a small quantity of Cx26 can maintain the cochlear physiology and the Cx26 expression in normal mature cochlea may have certain reserve capacity. However, the dose-effect relationship between Cx26 levels and the degree of hearing impairment and cell degeneration in mature cochleae needs further study.

Noise is one of the most prevalent ototoxic stresses and causes different patterns of hearing loss that are associated with cellular injury in accordance with the noise intensity and exposure time [30,31]. One hypothesis is that reduced Cx26 during mature stages may be a risk factor for specific types of deafness, especially noise-induce hearing loss. In our observations, a 13 dB gap was found at 8 kHz between the noise and KN group at P45. Moreover, reducing Cx26 resulted in greater OHC loss and the OC collapse, suggesting that the Cx26 loss exacerbates acoustic trauma and cell degeneration. It was reported that the outer hair cells in the basal turn were the most vulnerable targets for noise exposure and the Pillar and Deiter cells had more resistant ability for high intensity noise [30,32,33,34]. In our study, the OHC loss in noise group was the major pathological change and it confirmed the previous observations. However, loss of supporting cell in the KN group indicated that reduced Cx26 may induce both OHC and supporting cell degeneration during acoustic trauma. It has been shown that supporting cells may played a decisive role in SGN survival [35], potentially explaining why there was an obvious SGN degeneration in the basal turn of the KN group. As a result of Cx26 reduction and noise exposure in mature cochlea, our results provide direct evidence to support the involvement of Cx26 in NIHL. Considering the different protein functional defects that are caused by the various *GJB2* mutation genotypes, the human *GJB2* gene mutations/SNPs may have more complicated effects on NIHL than those observed herein. A transgenic mouse model, such as a mouse carrying the 35delG mutation, should be established for further investigation.

It is possible that the severe cell degeneration may be induced by the synergistic effects of decreased Cx26 and noise exposure in the KN group; however, the mechanisms still need to be investigated. It is thought that the gap junction can connect the adjacent supporting cells and that as a result, the energetic metabolites could be transmitted through it. In embryonic Cx26 null mice, Claudius cell degeneration was first found at P8. Shortly afterwards, dramatic OHC and surrounding supporting cell death were observed at approximately P13 [17,36]. Glucose imaging research has suggested that the glucose analogue distribution is depressed by uncoupling the gap junction in spiral limbus and spiral ligament [37,38]. Similarly, Lin’s group has found that glucose analogue among cochlear Claudius cells was severely reduced in Cx30 null mice [39]. Together, these results suggest that there may be aberrant energy metabolism in the mature supporting cells that lack the Cx26. Additionally, the noise exposure could also induce energy depletion in hair cells and supporting cells. The intracellular ATP concentrations in cochlear extracts have been shown to decrease immediately after noise exposure, and p-AMPKα, an energy sensor activated by an up-regulation in the AMP/ATP ratio, has also been shown to increase in the sensory hair cells of mice 1 h after traumatic noise exposure[40]. Further, Kiyokazu Ogita *et al.* [41] have observed that the p-AMPKα is elevated in Pillar cells and lateral wall structures after noise exposures of at least 110 dB. These data indicated that the combined effects of reduced Cx26 and noise exposure may induce excessive energy depletion and thereby result in cell degeneration. Moreover, in previous studies, it have been suggested that the generation of reactive oxygen species (ROS) may be an underlying cause of NIHL [42,43]. In cultured endothelial cells, localized oxidative insults have been shown to propagate between cells through gap junction (Cx43) inter-cellular communication [44]. In another study, lack of Cx43 or blockage of the Cx43 channel could increase ROS-induced astrocytic death [45]. In cochleae, the loss of Cx26 in the OC may cause dysfunction of the heterogenic Cx26/Cx30 gap junctions and obstructed the ROS propagation. Excessive ROS generation induced by the combined effects of noise exposure and Cx26 deletion may be the cause of severe cell death.

## 4. Materials and Methods

### 4.1. Generation of the Connexin26 (Cx26) Conditional Knockdown (KD) Mouse Model and Genotyping

Cx26^loxP/loxP^ mice and Rosa26CreER^T^ mice were kindly provided by Xi Lin at Emory University. Mice were raised in the specific-pathogen free (SPF) Experimental Animal Centre of Huazhong University of Science and Technology. As reported previously, Cx26^loxP/loxP^ mice were crossed with Rosa26CreER^T^ mice to generated Cx26^loxP/loxP^; Rosa26CreER^T^ mice [22]. The Cx26 in these mice could be deleted by tamoxifen induced Cre recombinase activation. Mouse genotyping was performed by PCR amplification of tail genomic DNA. The primer pairs for Cx26 floxed allele and Cre transgene were as follows: Cx26F: 5’-ACAGAAATGTGTTGGTGATGG-3’ and Cx26R: 5’-CTTTCCAATGCTGGTGGAGTG-3’; CreF: 5’-AGCTAAACATGCTTCATCGTCG GTC-3’and CreR: 5’-TATCCAGGTTACGGATATAGTTCATG-3’. In this study, all mice were injected tamoxifen (1.2 mg/10 g, T5648-1G, Sigma–Aldrich, St. Louis, MO, USA) intraperitoneally at P18. Animal experiments in this study followed protocols approved by the Institutional Animal Care and Use Committee at Tongji Medical College, Huazhong University of Science and Technology (Permit No. S429).

### 4.2. Animal Treatment and Noise Exposure Procedure

Mice were divided into four groups: control group, knockdown group (KD group), noise group and knockdown + noise group (KN group). Tamoxifen induced Cx26^loxP/loxP^; Rosa26CreER^T^ mice were used for KD and KN group, while the littermates lacking Cre were used for the control and noise group.

Mice in noise group or KN group were exposed to white noise at 110 dB SPL (sound pressure level) in a soundproof chamber. The noise exposure was imposed on mice at P25 and sustained 8 h per day for five consecutive days. A loudspeaker (TSM-102, Chanstek Audio Inc., Foshan, China) suspended 20 cm above the mouse cage was driven by a power amplifier (TMA-101, Tamo, HuaTai electronic Co., Hangzhou, China) that was fed from a computer. A PlayOne software was used to manipulate the exposure procedure. The mouse cage (290 × 190 × 225 mm) is made of polycarbonate with an iron railing on the roof and no more than 5 mice were loaded at once. Sound levels was calibrated by a sound level meter on the roof of the cage (TES1350A, TES Electrical Electronic Corp., Guangzhou, China) with about 2 dB SPL attenuation in four corners.

### 4.3. Auditory Brainstem Recordings

Auditory brainstem response was performed at P30 and P45. Mice (*n* = 6–12 in each group) were anesthetized with ketamine (120 mg/kg i.p.) and chlorpromazine (20 mg/kg, i.p.), and body temperature was maintained with a heating pad in a soundproof chamber. Recording electrode was placed at the vertex, and a reference electrode was inserted at tested ear with a ground electrode at the contralateral ear. Tone burst stimuli were generated and responds were recorded by the Tucker-Davis Technologies System (RZ6, Tucker-Davis Tech., Alachua, FL, USA) at frequencies of 8, 16, 24, 32 and 48 kHz. A loudspeaker (MF-1, Tucker-Davis Tech.) connected to the system was placed 10 cm away from the tested ear with another ear plugged. Responses were averaged 1024 times and recorded in decreasing 10 dB steps, then narrowing to 5 dB step near the threshold. The lowest sound level that elicited a repeatable wave was considered as the threshold.

### 4.4. Protein Extraction and Western Blot Analysis

The Cx26 protein levels (*n* = 4 in each group) were determined by western blot analysis. Cochleae were carefully dissected in ice cold PBS, and samples of membranous labyrinth were proceed in RIPA lysis buffer (P0013B, Beyotime Biotechnology, Haimen, China). A BCA Protein Assay Kit (P0012S, Beyotime) was used to determine the protein concentrations.

Proteins were separated by electrophoresis on 12% sodium dodecyl sulphate (SDS) polyacrylamide gels and then transferred to polyvinylidenedifluoride (PVDF) membranes. The membranes were blocked in TBST containing 5% milk for 1 h and then incubated overnight with rabbit polyclonal antibodies against Cx26 (1:1000, 710500, Invitrogen, Thermo Fisher Scientific Inc., Waltham, MA, USA) or rabbit polyclonal antibodies against β-actin (1:1000, 04-1116, Millipore, Merck Millipore Corporation, Darmstadt, Germany). Immunodetection was accomplished with horseradish peroxidase-conjugated goat anti-rabbit antibody and chemiluminescence with an ECL reaction kit (P0018, Beyotime). Bands were recorded by exposure on medical film and were analyzed by normalizing to the corresponding controls using Quantity One 4.6.2 Software (Bio-Rad Laboratories Inc., Alfred Nobel Drive Hercules, CA, USA).

### 4.5. Cochlear Tissue Preparation and Immunofluorescent Labeling

The temporal bones were harvested and dissected at P30 and kept in 4% paraformaldehyde in PBS for 1h at room temperature. After decalcification with disodium EDTA for 48–60 h, the cochleae were dehydrated with 20% and 30% sucrose for 1.5 h respectively and embedded in OCT. Modiolar sections with a thickness of 10 µm were cut for subsequent procedure. For flattened cochlear preparations, the apical basilar membrane was carefully dissected from fresh cochleae in 4% paraformaldehyde, and washed in 0.01 M PBS. The sections or flattened cochlear preparations were incubated and permeabilized in a blocking solution (10% donkey serum and 0.3% Trion X-100) for 1h at room temperature. Cx26 protein was recognized by overnight incubation at 4 °C with a polyclonal rabbit anti-Cx26 antibodies (1:200, Invitrogen) diluted in 0.01 M PBS. After washing with PBST (0.01 M PBS with 0.1% Tween-20), samples were stained by a fluorescently tagged secondary antibody (1:200, ANT030, Antegen Biotechnology Inc., Wuhan, China) for 1 h and DAPI (C1005, Beyotime Biotechnology, Haimen, China) for 5 min at room temperature, respectively. For flattened cochlear preparation immunostaining, the *F*-actin was additionally stained by phalloidin (0.005 mg/mL, P5282, Sigma, Sigma-Aldrich Corporation, St. Louis, MO, USA) for 30 min at room temperature. Images were captured on fluorescence microscope (DM2500, Leica, Leica Microsystems, Wetzlar, Germany). Images were post-processed with Adobe Photoshop software (Version 10.0, Adobe Systems Inc., San Jose, CA, USA).

### 4.6. Quantification of Cochlear Hair Cells

The flattened cochlear preparation (*n* = 3–4 in each group) dissected from decalcifying cochleae were separated into 4 portions. After permeabilization with 0.3% Triton X-100 in 0.01 MPBS for 5 min, the samples were stained by phalloidin (0.005 mg/mL, P1951, Sigma) for 30 min at room temperature. Nuclei were stained with DAPI for 5 min, and then the flattened preparations were mounted on glass slides. Images were taken with a laser confocal microscope (Nikon, Tokyo, Japan). To get the cochleograms, about 2100 outer hair cells in each sample were counted in 13 consecutive fields from the apex to the basal portion. The last part of the basilar membrane was anfractuous and hard to obtain, so the counted parts covered nearly 90 percents of the whole basilar membrane.

### 4.7. Resin Sections and Transmission Electron Microscopy

The cochleae (*n* = 3 in each group) were opened in the apex and flushed using 2% PFA with 2% glutaraldehyde in 0.1 M PB buffer. After decalcification, the samples were post-fixed for one hour in 1% osmium tetroxide and then dehydrated through a graded ethanol series. After embedding using an Epon812 kit, cochleae were sectioned (2 µm in thickness) and stained with toluidine blue for light microscope observation. The ultrathin sections (80 nm in thickness) were stained with uranyl acetate and lead citrate for electron microscopy examination (H7100, Hitachi, Tokyo, Japan).

### 4.8. Statistical Analysis

All data were presented as mean ± s.e.m. and plotted by SigmaPlot (Version 12.5, Systat Software, Inc., San Jose, CA, USA). One-way ANOVA with a LSD correction or *t*-test were performed by SPSS software (version 19, IBM SPSS Statistics, New York, NY, USA) for statistical analysis. *p* < 0.05 was considered to be statistically significant.

## 5. Conclusions

In conclusion, the present study demonstrates that mature cochleae (P18) with partial loss of Cx26 did not exhibit auditory dysfunction and cell degeneration in a certain period. However, reduced Cx26 in mature cochleae in mice increased susceptibility to noise-induced hearing loss.

## Supplementary Materials

Supplementary materials can be found at http://www.mdpi.com/1422-0067/17/3/301/s1.

## Abbreviations

Cx26: connexin26
NIHL: noise-induced hearing loss
KD: knocked down
GJ: gap junction
SNPs: single nucleotide polymorphisms
SPF: specific-pathogen free
KN: knocked down + noise
HC: hair cell
OHC: outer hair cell
SV: stria vascularis
TC: tunnel of Corti
MC: marginal cells
OC: organ of Corti
BC: basal cell
