# Supplementary material for: Reduced Connexin26 in the Mature Cochlea Increases Susceptibility to Noise-Induced Hearing Loss in Mice
Source: Int J Mol Sci. 2016 Feb 26;17(3):301. doi: 10.3390/ijms17030301 (PMC4813165; doi:10.3390/ijms17030301)
Supplement: Supplementary file 1 [file ijms-17-00301-s001.pdf]

Supplementary Materials: Reduced Connexin26 in the Mature Cochlea Increases Susceptibility to Noise-Induced Hearing Lossin Mice

Xing-Xing Zhou, Sen Chen, Le Xie, Yu-Zi Ji, Xia Wu, Wen-Wen Wang, Qi Yang, Jin-Tao Yu, Yu Sun, Xi Lin and Wei-Jia Kong

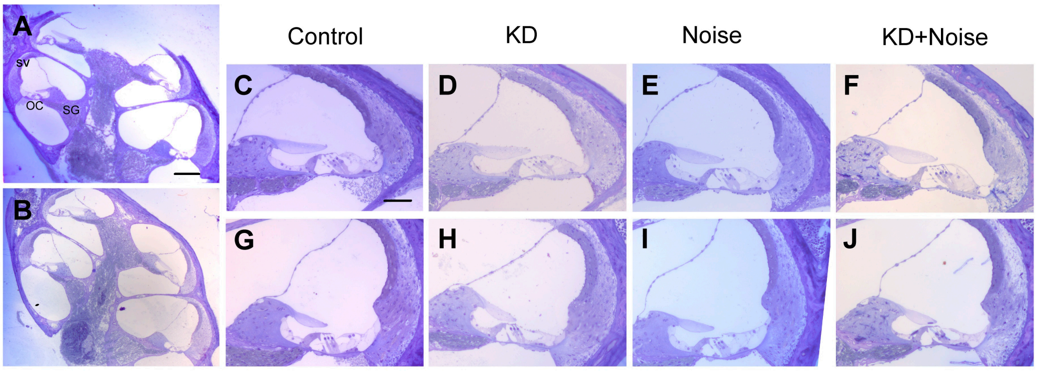

Figure S1. Cochlear morphology of the apical and middle turn in control, KD, noise and KN groups. A full view of a cochlea obtained from control (**A**) and KD group (**B**); Panels **C**, **D**, **E** and **F** show the morphology of the apical cochleae in control and experimental groups; Panels **G**, **H**, **I** and **J** show the middle cochleae in the control and experimental groups, respectively. The scale in panel **A** represents 200 μm, and scale in panel **C** represents approximately 40 μm. Abbreviations: SV: stria vascularis; OC: organ of Corti; SG: spiral ganglia.
